# Classic Publications in the Field of Dentistry: A Bibliometric Analysis

**DOI:** 10.1016/j.identj.2025.100909

**Published:** 2025-07-17

**Authors:** Yuh-Shan Ho, Nikolaos Christidis

**Affiliations:** a CT HO Trend, Taipei City, Taiwan; b Division of Oral Rehabilitation, Department of Dental Medicine, Karolinska Institutet, Huddinge, Sweden

**Keywords:** Dentistry, Oral surgery and medicine, Classic articles, SCI-EXPANDED, Scientometrics

## Abstract

**Introduction and aims:**

This bibliometric study aimed to identify and analyze the classic articles in the Web of Science category ‘Dentistry, Oral Surgery and Medicine,’ providing insights into the characteristics, citation patterns, and evolving trends that define the field's most influential publications.

**Methods:**

Articles published between 1960 and 2020 were extracted from the Science Citation Index Expanded (SCI-EXPANDED) database. Classic articles were defined as those with ≥1000 citations as of the end of 2024. Bibliometric indicators such as total citations (*TC_2024_*), citations in 2024 (*C_2024_*), citations per publication (*CPP_2024_*), and authorship patterns were analyzed. Citation trajectories, author keywords, and institutional and international collaborations were also assessed.

**Results:**

A total of 42 classic articles were identified. These publications were most frequently produced in the 1990s and 2000s and originated predominantly from the USA and Sweden. The University of North Carolina and the University of Gothenburg were particularly prominent. Periodontology, implantology, dental materials, and oral epidemiology were common themes. Articles such as Socransky et al. and Schiffman et al. demonstrated both sustained and recent citation influence. Word frequency analysis highlighted topics such as plaque, periodontal disease, and classification systems. Citation histories revealed diverse trajectories, including delayed recognition and rapid recent influence.

**Conclusion:**

Classic articles in dental research reflect the field’s shift toward evidence-based practice, interdisciplinary collaboration, and public health relevance. By mapping these highly cited works, this study provides a clearer understanding of the factors contributing to long-term scientific impact in dentistry, oral surgery, and medicine.

**Clinical relevance:**

This study identifies highly cited publications that have shaped clinical practice in periodontology, implantology, temporomandibular disorders, wound healing, and oral-systemic health. These works continue to guide diagnostics, treatment planning, and public health strategies, highlighting the lasting impact of foundational research and the importance of interdisciplinary, evidence-based approaches in dentistry.

## Introduction

There is a strong desire among researchers in the field of dentistry, as for researchers in other medical and scientific disciplines, to share their research findings with a broad audience that includes other researchers, clinicians, students, patients, and policy-makers. Through this spread of research findings, there is hope that new knowledge can contribute to a deeper understanding of disease mechanisms, diagnostic and therapeutic methods, clinical decision-making, and ultimately patient care and oral health outcomes.^1^, ^2^, ^3^ This is particularly important in dentistry, oral surgery, and oral medicine, which together encompass a wide spectrum of conditions and procedures, ranging from preventive strategies and caries management to implantology, oral pathology, and maxillofacial surgery. Research in this field addresses not only biological and clinical aspects, but also behavioral, public health, and systemic factors influencing oral health.^3^

In order to evaluate whether scientific findings in this broad and clinically important area have had an impact (whether on further research, education, clinical guidelines, or health policy), it is necessary to apply scientometric methods. Scientometrics, or bibliometrics as it is also called, has been widely used since the 1960s to quantify and visualize the scientific impact of publications.^4^ One such method is citation analysis, which examines how often a publication has been cited by others. Although citations are not a direct measure of scientific quality, they can indicate the influence, recognition, and popularity of a publication or its topic within its field.^5^, ^6^, ^7^ Thus, highly cited papers are often referred to as "classic articles" and can be seen as milestones that have shaped the scientific and clinical development of a field.

There are many scientific and clinical subfields within “Dentistry, Oral Surgery and Oral Medicine,” and the most cited articles often reflect important shifts in knowledge, clinical practices, or methodological breakthroughs. For example, Socransky et al. introduced the concept of microbial complexes in subgingival plaque, laying the foundation for our understanding of the microbial etiology of periodontal disease.^8^ Similarly, Brånemark's work on osseointegration transformed implant dentistry into a predictable clinical discipline,^9^ Guo and DiPietro’s review on wound healing mechanisms provided critical insights into factors influencing oral and peri-implant tissue repair,^10^ and Schiffman et al. who introduced the new diagnostic criteria for temporomandibular disorders (TMD) for both clinicians and research that are worldwide accepted and used.^11^ More recently, Xu et al. demonstrated high angiotensin-converting enzyme II (ACE2) receptor expression in oral mucosa, a finding that gained enormous significance during the COVID-19 pandemic as it highlighted the oral cavity as a potential transmission route for SARS-CoV-2.^12^ Articles such as these represent not only high scientific value but also societal relevance. Classic publications such as the gingival index by Löe et al. and the plaque and gingivitis scoring system proposed by Ainamo and Bay are further examples of diagnostic and epidemiological tools that have had enduring influence.^13^^,^^14^ The continued citation of these works decades after their publication illustrates their foundational role in oral health research and practice.

By systematically identifying and analyzing the most cited publications in this domain, we gain important insights into the trajectory of dental science, the dissemination of key findings, and the evolving priorities of the research community.

In 2011, Ho’s research team introduced a more robust citation indicator known as TC_year_, which quantifies the total number of citations a publication has received in the Web of Science Core Collection (WoSCC) from its publication year through to the end of a designated, most recent year.^6^^,^^15^ Unlike raw citation counts that are subject to change over time, TC_year_ remains constant, offering a stable and reproducible measure for bibliometric analysis.^16^ Based on this methodology, publications with TC_year_ ≥ 1000 citations have been classified as “classic” works.^17^ This metric has since been widely utilized across various medically oriented WoS categories, including psychology,^5^ neurosciences,^18^ orthopedics,^19^ and obstetrics and gynecology.^20^

In 2012, Ho further proposed the Y-index, a bibliometric tool designed to assess the scholarly impact of authors by focusing on their number of first-author and corresponding-author publications with high citation counts. The Y-index consists of 2 components: j, which reflects the overall publication potential, and h, which characterizes the type of contribution. This dual-parameter metric offers a nuanced assessment of an author’s research influence.^4^^,^^21^ The Y-index has been applied to evaluate the output of countries, institutions, and individual scholars.^4^ More recently, it has been employed to compare prominent authors in various SCI-EXPANDED medical categories, such as emergency medicine,^22^ anesthesiology,^23^ ophthalmology,^24^ and tropical medicine.^25^

The present study aimed to identify and analyze the “classic” publications in the Web of Science Category (WoSCC) of “Dentistry, Oral Surgery and Medicine” within the SCI-EXPANDED. In the present study, publications were identified as “classic” if they accrued 1000 or more citations in the WoSCC from the year of publication through the end of 2024. Basic bibliometric metrics were computed using Microsoft Excel. Furthermore, the Y-index was employed to evaluate the research productivity of authors of these classic publications.

## Methodology

The data utilized in this study were obtained from the Web of Science Core Collection (WoSCC) provided by Clarivate Analytics, specifically from the online version of the Science Citation Index Expanded (SCI-EXPANDED), with the dataset last updated on 2 June 2025. As of that date, SCI-EXPANDED indexed 9486 journals covering 178 Web of Science categories. Of these, 91 journals were categorized under ‘dentistry, oral surgery & medicine’ in the Web of Science classification.

Publications were assessed using following citation indicators:

C_year_: the number of citations from Web of Science Core Collection in a particular year (e.g. C_2024_ describes citation count in 2024).^4^
TC_year_: the total citations from Web of Science Core Collection received since publication year till the end of the most recent year (2024 in this study, TC_2024_).^6^
CPP_year_: average number of citations per publication (CPP_2024_ = TC_2024_/TP), TP: total number of publications.^26^

The use of C_year_, TC_year_, CPP_year_ offers distinct advantages due to their invariance and reproducibility, in contrast to citation counts directly retrieved from the WoSCC, which are subject to ongoing changes.^5^ In this study, publications in the Web of Science category of ‘dentistry, oral surgery and medicine’ with a TC_2024_ of 1,000 or more were designated as classic publications.^17^

A total of 64 documents with a *TC* (total citations from Web of Science Core Collection) of 1,000 or more citations were searched-out in SCI-EXPANDED from 1964 to 2020. For each document, the full bibliographic records and annual citation data were downloaded into Microsoft Excel 365, and further manual coding was conducted.^27^ Several Excel functions, such as COUNTA, CONCATENATE, FILTER, MATCH, VLOOKUP, PROPER, RANK, REPLACE, FREEZE PANES, SORT, SUM, and LEN, were utilized for data processing and analysis.^28^

After thorough validation, 63 documents with a verified TC_2024_ ≥ 1000 citations (representing 98% of the initially retrieved 64 documents) from 1964 to 2020 were formally classified as classic publications in this field. The Journal Impact Factors (IF_2023_) were obtained from the 2024 edition of the Journal Citation Reports (JCR).

For additional accuracy, hard copies of 42 classic articles indexed under the “dentistry, oral surgery and medicine” category in SCI-EXPANDED were collected to verify author information. In cases where the corresponding author was not explicitly indicated, the first author was assumed to hold this role. Furthermore, institutional affiliations were standardized: those from England and Wales were grouped under the United Kingdom (UK), and affiliations listed as Fed Rep Ger (Federal Republic of Germany) were reclassified under Germany.^4^

To provide a more comprehensive evaluation of research output, 6 publication indicators were introduced in 2014 to assess the performance of countries and institutions.^29^^,^^30^ These indicators include:

• TP: Total number of classic articles published.
• IP: Number of classic articles published by a single country (IP_C_) or single institution (IP_I_).
• CP: Number of internationally collaborative classic articles (CP_C_) or inter-institutionally collaborative classic articles (CP_I_).
• FP: Number of classic first-author articles.
• RP: Number of classic corresponding-author articles.
• SP: Number of classic single-author articles.

Moreover, 6 corresponding citation indicators, expressed as CPP_2024_ (average number of citations per publication), were employed to evaluate the impact of publications by document type, journal, country, and institution, as proposed by Ho and Mukul in 2021.^31^ These citation indicators correspond directly to the 6 previously established publication indicators, thereby enabling a comprehensive assessment of both research productivity and citation impact.

The Y-index was utilized to assess the publication performance of authors, following the methodology originally proposed by Ho in 2012 and 2014.^4^^,^^21^ The Y-index is defined as follows: Y-index (j, h), where: j is a constant representing the publication potential of an author, calculated as the sum of classic first-author (FP) and classic corresponding-author (RP) articles h is the angular component (in radians), indicating the author’s publication characteristics based on the proportion of RP to FP.

A higher value of j indicates greater overall contribution as a first and/or corresponding author. The value of h provides insight into the nature of the author’s contributions:

• h = π/2: the author has published only corresponding-author articles (j = RP);
• π/2 > h > π/4: the author has more corresponding-author articles than first-author articles (FP > 0);
• h = π/4: the author has an equal number of first-author and corresponding-author articles (FP > 0 and RP > 0);
• π/4 > h > 0: the author has more first-author articles than corresponding-author articles (RP > 0);
• h = 0: the author has published only first-author articles (j = FP).

This 2-dimensional indicator offers a nuanced view of authorship roles and contribution patterns in scholarly publications.

## Results and discussion

### Document type and language of publication

To examine the characteristics of classic document types within a specific Web of Science category, key bibliometric indicators, such as the average number of citations per publication (CPP_year_) and the average number of authors per publication (APP), have been proposed and applied in previous studies.^18^

In this study, a total of 63 classic documents in the ‘dentistry, oral surgery and medicine’ category of the SCI-EXPANDED database was identified, each receiving 1000 or more citations (TC_2024_). Supplementary table 1 summarizes the characteristics of the 6 identified document types, including the total number of publications in a document type (TP), APP, and CPP_2024_ for each type.^32^

Among these, articles were the most dominant document type, accounting for 42 records (67%) of the 63 classic publications, with an average of 5.9 authors per article. The highest CPP_2024_ was observed in the meeting abstract category, which, despite consisting of only a single document, achieved an exceptionally high CPP_2024_ of 2640. This outlier is attributed to the classic meeting abstract titled “The Gingival Index, the Plaque Index and the Retention Index Systems” by Löe^33^ which alone accumulated 2,640 citations by the end of 2024. The exceptionally high citation impact of this meeting abstract^33^ underscores its foundational significance in periodontal research. Despite being a brief congress abstract rather than a full-length peer-reviewed article, its indices have profoundly influenced periodontal diagnostics and clinical research practices. Particularly in the past 2 decades, the continued relevance of these indices is due to their robustness, simplicity, and widespread adoption in clinical trials and epidemiological studies. These characteristics have allowed consistent cross-study comparisons, greatly facilitating standardized periodontal assessments. Recent significant works that have employed these indices include for instance studies with revised criteria for evaluating direct and indirect dental restorations,^34^ disease progression in periodontitis over time (40 years),^35^ teledentistry and prevention of oral health,^36^ and evaluating peri-implant tissue health and implant failure.^37^ The enduring utility and simplicity of Löe’s guides continue to cement their central role in contemporary periodontal diagnostics and preventive strategies.

The only letter among the classic documents was titled “Pamidronate (Aredia) and Zoledronate (Zometa) Induced Avascular Necrosis of the Jaws: A Growing Epidemic” by Marx, published in the Journal of Oral and Maxillofacial Surgery.^38^ This letter received a TC_2024_ of 2016 citations, highlighting its significant impact despite its brief format. It is 1 of the most impactful clinical alerts in the field of oral and maxillofacial surgery was published as a letter to the editor by Marx in 2003, warning of a previously unrecognized adverse effect of intravenous bisphosphonate therapy, that is avascular necrosis of the jaws.^38^ Although the format was a brief preliminary report rather than a full peer-reviewed study, the communication gained substantial traction due to its timely recognition of a serious and growing clinical issue. It documented 36 cases of nonhealing exposed bone in patients treated with pamidronate or zoledronate, highlighting the unique vulnerability of the jawbones to bisphosphonate-induced osteonecrosis, likely due to the high bone turnover and frequent dental interventions in this anatomical region.

The impact of this report in the past 2 decades has been profound. It not only triggered a cascade of clinical investigations and mechanistic studies, but also reshaped practice guidelines across oncology, dentistry, and surgery.^39^, ^40^, ^41^ The phenomenon, later termed medication-related osteonecrosis of the jaw (MRONJ) or bisphosphonate-related osteonecrosis of the jaws (BRONJ), led to significant changes in drug warnings, risk assessment protocols, and patient management strategies.^42^^,^^43^ The report's enduring influence lies in its role as the catalyst for recognition, classification, and preventive guidelines for MRONJ/BRONJ, a condition that continues to be a challenge in dental and surgical care for cancer patients.

The only editorial material among the classic publications was titled “Peri-implant Diseases: Consensus Report of the Sixth European Workshop on Periodontology” by Lindhe and Meyle, published in the Journal of Clinical Periodontology.^44^ This editorial received a TC_2024_ of 1,081 citations, reflecting its influence as a key consensus document in the field. This editorial is also influential publication in the field of periodontal and implant research and is a consensus report authored by Lindhe and Meyle on behalf of Group D of the Sixth European Workshop on Periodontology. Although published in the format of a letter-style consensus summary, the report has served as a landmark document by providing standardized definitions and diagnostic criteria for peri-implant diseases, including peri-implant mucositis and peri-implantitis. It also highlighted prevalence data and established associations with key risk indicators such as poor oral hygiene, a history of periodontitis, smoking, and diabetes. The clinical importance of this report lies in its contribution to establishing an international consensus on how to diagnose, prevent, and manage peri-implant diseases. The report emphasized the limitations of non-surgical treatments for peri-implantitis, the unpredictable nature of current therapies, and the necessity of including anti-infective measures in both non-surgical and surgical approaches. Since its publication, the report has been extensively cited and incorporated into global clinical guidelines, influencing treatment planning and follow-up protocols in implant dentistry.^45^, ^46^, ^47^, ^48^, ^49^ Its lasting impact is also reflected in how it laid the foundation for subsequent research exploring the epidemiology, risk assessment, and long-term management of peri-implant conditions. The report remains 1 of the most authoritative references in the domain of peri-implant disease, frequently used to benchmark clinical trials and guide both academic curricula and everyday clinical protocols.^45^, ^46^, ^47^, ^48^, ^49^

Review papers exhibited a *CPP*_2024_ that was 1.1 times higher than that of articles, indicating a modestly greater citation impact. This contrasts with findings in the Web of Science category of neurosciences, where review papers had a CPP_year_ only 0.95 times that of articles,^18^ suggesting that in the field of dentistry, oral surgery and medicine, reviews may hold relatively higher influence.

A total of 18 classic review articles were published across 12 journals within the “dentistry, oral surgery and medicine” category in SCI-EXPANDED. The majority appeared in the Journal of Dental Research, which holds a 2023 impact factor (IF_2023_) of 5.7 and ranks 4^th^ among 91 journals in this category. This journal published 5 reviews (28% of the 18), with an average CPP_2024_ of 1,712 citations per publication and an APP of 4.8 authors per review.

Among the 63 classic documents identified in this category, 18 were reviews. The review titled “Factors Affecting Wound Healing” by Guo and DiPietro was the most frequently cited,^10^ with a TC_2024_ of 3351 citations. This article comprehensively examines wound healing, a complex process involving 4 key phases: hemostasis, inflammation, proliferation, and remodeling. It highlights how factors such as infection, oxygen availability, age, stress, and lifestyle can impede healing, and explores underlying biological mechanisms to guide the development of improved therapeutic approaches. Additionally, this review was the most impactful in 2024 within the category, accumulating 449 citations in that year alone (C_2024_). Its continued influence in recent years can be attributed not only to the clarity of its synthesis but also to its broad applicability across multiple fields, including oral surgery, periodontology, implantology, and regenerative dentistry. Guo and DiPietro’s review provided a conceptual framework for how local and systemic conditions alter the biological wound healing cascade, a topic that is directly relevant to both surgical outcomes and tissue integration in dentistry. In particular, the article has been frequently cited in studies evaluating peri-implant healing, guided tissue regeneration, and soft tissue management.^50^, ^51^, ^52^ For example, it has informed the understanding of impaired healing in patients with diabetes or smokers undergoing periodontal or implant surgery, as well as the design of bioactive materials aimed at modulating inflammatory and proliferative phases.^50^^,^^53^, ^54^, ^55^ Its role in connecting cellular mechanisms with clinical implications has made it a foundational reference in translational dental research.

For subsequent analyses, only articles were included, as they generally offer comprehensive content encompassing an introduction, methodology, results, discussion, and conclusion.^56^ A total of 42 classic articles, all published in English, were selected for further in-depth examination.

### Publication distribution

Ho’s research group proposed a unique bibliometric indicator that relates the total number of classic articles published in a decade (TP) to their average number of citations per publication (CPP_year_) within a given Web of Science category.^17^ In the category of ‘dentistry, oral surgery and medicine’, the 42 classic articles identified were published between 1964 and 2020, accumulating a combined TC_2024_ of 1,736,344 citations. Citation counts for these articles ranged from a maximum of 3470 to a minimum of 1007 citations, with an average of 1457 citations per article.

The earliest classic article, published in 1964, was titled “The Simplified Oral Hygiene Index” by Greene and Vermillion.^57^ This work introduced the Simplified Oral Hygiene Index (OHI-S), a streamlined version of the original Oral Hygiene Index designed for rapid assessment of oral cleanliness in population groups. Although slightly less sensitive than the original index, the OHI-S provided a practical and efficient tool for large-scale epidemiological studies. The most recent classic article was published in 2020: “High expression of ACE2 receptor of 2019-nCoV on the epithelial cells of oral mucosa” by Xu et al.^12^ This article, which received a TC_2024_ of 1754 citations, ranked seventh in the dataset. During the COVID-19 pandemic, Xu and colleagues discovered that the ACE2 receptor, critical for SARS-CoV-2 entry, is highly expressed in the oral mucosa, particularly in the tongue’s epithelial cells, highlighting the oral cavity as a potential high-risk site for viral infection.

**Fig. 1:**
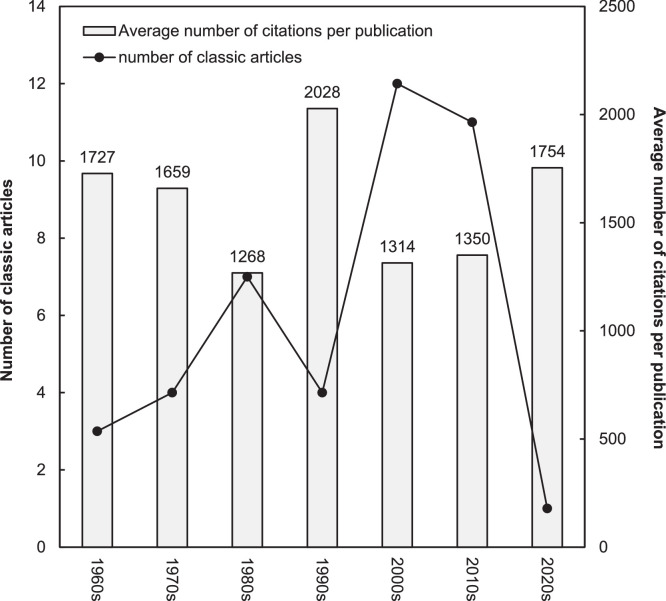
Number of classic articles and average number of citations per publication by decade.

Figure 1 illustrates the distribution of these 42 classic articles across decades alongside their CPP_2024_ values. The decade with the highest number of classic articles was the 2000s, accounting for 12 publications (29% of the total). It is widely recognized that recent articles require time to accumulate citations, which explains the observed trend that it took over a decade to reach the peak number of classic articles in this category. Comparable patterns have been reported in the Neurosciences category.^18^ In contrast, other fields such as orthopedics^19^ and psychology,^5^ as well as surgery,^17^ required 2 to 3 decades to reach their peak classic article counts.

When an article attains classic status within a decade of its publication, it is considered particularly distinguished in the **“**dentistry, oral surgery and medicine” category. Examples include works by Xu et al.^12^ Schiffman et al.^11^ and Ruggiero et al.^43^ The year 2018 was the most productive, with 4 classic articles published. No classic articles have been identified from before 1964 or after 2020.

Among these, Schiffman et al. introduced the evidence-based Diagnostic Criteria for Temporomandibular Disorders (DC/TMD) protocol, a standardized and validated diagnostic tool for TMD. This protocol enhances diagnostic accuracy, supports early detection and classification, and improves consistency in both clinical practice and research settings.^11^

The 1990s, with 4 classic articles, recorded the highest CPP_2024_ at 2028 citations per publication. This peak can be attributed in part to 3 of the top ten most cited articles, all published during this decade: Socransky et al. with 3470 citations (ranked 1^st^),^8^ Marx et al. with 1929 citations (ranked 6^th^),^58^ and Slade with 1678 citations (ranked 8^th^).^59^ Based on this, the 1990s seem to stand out as a particularly impactful decade in the “Dentistry, Oral Surgery and Oral Medicine” category, as evidenced by the high *CPP_2024_* and the presence of several influential publications. The 3 above mentioned among the top ten most cited articles (Socransky et al. Marx et al. and Slade^8^^,^^58^^,^^59^) underscore key advances in periodontal microbiology, regenerative surgery, and oral health-related quality of life, respectively. In the study by Socransky et al. a pivotal cluster-based classification of subgingival plaque microbial complexes was introduced. This classification transformed the understanding of periodontal pathogenesis by grouping bacteria into color-coded complexes, notably the “red complex” linked to disease severity. This classification continues to guide both diagnostics and periodontal therapy.^8^ In parallel, the study by Marx et al. provided 1 of the earliest and most influential demonstrations of the clinical utility of platelet-rich plasma (PRP) in oral and maxillofacial bone grafting. By enhancing bone regeneration through autologous growth factor delivery, this study laid the groundwork for the now widely accepted use of PRP in implantology and reconstructive procedures.^58^ Finally, the study by Slade contributed to a different but equally important advancement with the development and validation of the short-form Oral Health Impact Profile (OHIP-14), a psychometrically sound and widely adopted instrument for assessing oral health-related quality of life. This tool enabled a broader integration of patient-centered outcomes in both clinical trials and population-based studies.^59^ Together, these 3 articles not only exemplify methodological innovation and clinical applicability but also reflect the broader maturation of dental research in the 1990s, that is from basic science to translational and patient-centered outcomes. Their continued influence, as shown by high recent citation counts, attests to their foundational role in shaping contemporary practice and research.

### Journals in ‘dentistry, oral surgery and medicine’ category in SCI-EXPANDED

The 42 classic articles in the “dentistry, oral surgery and medicine” category were published across 21 journals indexed in SCI-EXPANDED in 2023. Four of these journals were not listed under this category in 2023 including Oral Surgery Oral Medicine Oral Pathology Oral Radiology and Endodontology published 4 classic articles, Oral Surgery Oral Medicine Oral Pathology Oral Radiology and Endodontics, Dental Clinics of North America, and Journal of Oral Surgery each published 1 classic article.

Thirteen journals published only a single classic article. Table 1 compares the top 8 most productive journals, each with 2 or more classic articles in the category. Among them, the *Journal of Clinical Periodontology*, with an IF_2023_ of 5.8 (ranked 3^rd^ out of 91 journals), published 6 classic articles. In terms of authorship patterns, the Journal of Oral & Facial Pain and Headache had the highest average number of authors per publication (APP = 34 for its single classic article), whereas the American Journal of Orthodontics and Dentofacial Orthopedics, Dental Clinics of North America, and Journal of Prosthetic Dentistry each published a single-author classic article.

**Table 1:** The top 8 productive journals with 2 or more classic articles in “dentistry, oral surgery and medicin” category in SCI-EXPANDED.

Journal	TP (%)	IF_2023_ (R)	APP	CPP_2024_
Journal of clinical periodontology	6 (14)	5.8 (3)	12	1662
Journal of dental research	5 (12)	5.7 (4)	7.6	1191
Journal of periodontology	5 (12)	4.2 (11)	4.2	1512
Oral surgery oral medicine oral pathology oral radiology and endodontology	4 (10)	1.457*	3.5	1249
Journal of oral and maxillofacial surgery	3 (7.1)	2.3 (42)	5.0	1385
Clinical oral implants research	2 (4.8)	4.8 (7)	5.0	1069
Community dentistry and oral epidemiology	2 (4.8)	1.8 (62)	1.0	1647
Dental materials	2 (4.8)	4.6 (9)	2.0	1318

TP, total number of classic articles; %, percentage of articles in all 42 classic articles; IF_2023_, journal impact factor in 2023;

⁎ journal impact factor in 2011 (IF_2011_); R, rank in 91 journals listed in ‘dentistry, oral surgery and medicine’ category in SCI-EXPANDED in 2023; APP, average number of authors per publication; CPP_2024_, average number of per publication (TC_2024_/TP).

The multi-author classic article titled “Diagnostic criteria for temporomandibular disorders (DC/TMD) for clinical and research applications: Recommendations of the international RDC/TMD consortium network and orofacial pain special interest group” involved 34 authors from 25 institutions across 12 countries.^11^ Schiffman et al. emphasized that while the original RDC/TMD Axis I algorithms were reliable, they lacked sufficient validity. This led to the development of revised, more accurate diagnostic tools. Axis II instruments were confirmed as valid and dependable. These findings informed international consensus workshops that finalized updated, validated Axis I and II tools for improved TMD diagnosis and assessment.^11^

Comparing the journals in Table 1, the Journal of Clinical Periodontology also had the highest *CPP*_2024_, averaging 2225 citations per publication from its 6 classic articles. The Clinical Oral Implants Research journal, with an IF_2023_ of 4.8 (ranked 7^th^), had a lower *CPP*_2024_ of 1069 citations per publication. The International Journal of Oral Science, with a single classic article, had the highest IF_2023_ of 10.8, ranking 2^nd^ among the 91 journals in the category.

### Countries, institutions, and authors

Ho and Mukul introduced 6 publication indicators and corresponding citation indicators to compare research performance at country and institutional levels.^31^ These indicators have since been applied in various fields, including tropical medicine^25^ and machine learning research.^60^

All 42 classic articles were manually checked for authorship information. Researchers affiliated with 19 different countries contributed to these articles. Among these: 27 articles (64%) were single-country publications from 7 countries, with a CPP_2024_ of 1465 citations per publication and 15 articles (36%) were internationally collaborative, involving co-authors from 19 countries, showing a slightly lower CPP_2024_ of 1442 citations per publication. This suggests that in “dentistry, oral surgery and medicine” research, international collaboration may be associated with a modestly lower citation impact for classic articles.

To compare countries’ research output, the 6 publication indicators and their corresponding citation indicators (CPP_2024_) proposed by Ho and Mukul were applied.^31^ Table 2 summarizes data for all 19 countries publishing classic articles. This list includes: 11 European countries, 4 from Asia, 3 from the Americas, and 1 from Oceania. No classic articles originated from Africa. Only 5 countries (USA, UK, Sweden, Switzerland, and Canada) had single-author classic articles. Seven, eleven, and eleven countries contributed single-country articles, first-author articles, and corresponding-author articles, respectively. These findings suggest that, in the field of “dentistry, oral surgery and medicine” research, international collaboration may be associated with lower citation impact in classic articles. To compare country-level research performance, 6 publication indicators and their corresponding citation indicators (CPP_2024_) were applied, as previously proposed by Ho and Mukul in 2021.^31^

**Table 2:** Comparison of publication performance of countries.

Country	TP	TP (n = 42)		IP_C_ (n = 27)		CP_C_ (n = 15)		FP (n = 42)		RP (n = 42)		SP (n = 7)	
		R (%)	CPP_2024_	R (%)	CPP_2024_	R (%)	CPP_2024_	R (%)	CPP_2024_	R (%)	CPP_2024_	R (%)	CPP_2024_
USA	29	1 (69)	1444	1 (63)	1.491	1 (80)	1376	1 (57)	1493	1 (55)	1507	1 (57)	1290
UK	9	2 (21)	1348	2 (11)	1.122	2 (40)	1461	2 (10)	1106	2 (12)	1115	2 (14)	1171
Sweden	7	3 (17)	1447	5 (3.7)	1.104	2 (40)	1505	4 (4.8)	1084	4 (4.8)	1084	2 (14)	1324
Switzerland	6	4 (14)	1432	3 (7.4)	1.325	4 (27)	1485	3 (7.1)	1219	3 (7.1)	1219	2 (14)	1615
Germany	5	5 (12)	1540	5 (3.7)	1.592	4 (27)	1527	7 (2.4)	1592	7 (2.4)	1592	N/A	N/A
Australia	4	6 (10)	1498	N/A	N/A	4 (27)	1498	N/A	N/A	N/A	N/A	N/A	N/A
Canada	4	6 (10)	1673	N/A	N/A	4 (27)	1673	7 (2.4)	1324	7 (2.4)	1324	2 (14)	1324
China	4	6 (10)	1429	5 (3.7)	1754	8 (20)	1320	4 (4.8)	1636	4 (4.8)	1636	N/A	N/A
Denmark	4	6 (10)	2164	3 (7.4)	1.870	11 (13)	2459	4 (4.8)	1870	4 (4.8)	1870	N/A	N/A
Brazil	3	10 (7.1)	1270	N/A	N/A	8 (20)	1270	7 (2.4)	1475	7 (2.4)	1475	N/A	N/A
Spain	3	10 (7.1)	1169	N/A	N/A	8 (20)	1169	N/A	N/A	N/A	N/A	N/A	N/A
Belgium	2	12 (4.8)	1832	N/A	N/A	11 (13)	1832	N/A	N/A	N/A	N/A	N/A	N/A
France	2	12 (4.8)	1996	N/A	N/A	11 (13)	1996	7 (2.4)	1393	7 (2.4)	1393	N/A	N/A
Italy	2	12 (4.8)	1935	N/A	N/A	11 (13)	1935	N/A	N/A	N/A	N/A	N/A	N/A
Netherlands	2	12 (4.8)	1935	N/A	N/A	11 (13)	1935	N/A	N/A	N/A	N/A	N/A	N/A
Finland	1	16 (2.4)	2319	N/A	N/A	16 (6.7)	2319	7 (2.4)	2319	7 (2.4)	2319	N/A	N/A
Israel	1	16 (2.4)	1271	N/A	N/A	16 (6.7)	1271	N/A	N/A	N/A	N/A	N/A	N/A
South Korea	1	16 (2.4)	1064	N/A	N/A	16 (6.7)	1064	N/A	N/A	N/A	N/A	N/A	N/A
Turkey	1	16 (2.4)	1271	N/A	N/A	16 (6.7)	1271	N/A	N/A	N/A	N/A	N/A	N/A

TP, number of total classic articles; TP R (%), total number of articles and the percentage of total 42 classic articles; IP_C_ R (%), rank and percentage of single-country articles in all 27 single-country articles; CP_C_ R (%), rank and percentage of internationally collaborative articles in all 15 internationally collaborative articles; FP R (%), rank and the percentage of first-author articles in all 42 first-author articles; RP R (%), rank and the percentage of corresponding-author articles in all 42 corresponding-author articles; SP R (%), rank and the percentage of single-author articles in all 7 single-author articles; CPP_2024_, average number of citations per publication (CPP_2024_ = TC_2024_/TP); N/A, not available.

The USA dominated all 6 publication indicators:

• Total Publications (TP): 29 classic articles (69% of 42 classic articles).
• Independent Publications (IP_C_): 17 articles (63% of 17 single-country classic articles).
• Collaborative Publications (CP_C_): 12 articles (80% of 15 internationally collaborative classic articles).
• First-Author Publications (FP): 24 articles (57% of 42 first-author classic articles).
• Corresponding-Author Publications (RP): 23 articles (55% of 42 corresponding-author classic articles).
• Single-Author Publications (SP): 4 classic articles (57% of 7 single-author classic articles).

Among the 19 countries, Finland (with 1 classic article) had the highest CPP_2024_ at 2319 citations per publication (total, first-author, and corresponding-author article). Denmark, with an IP_C_ of 2 articles and a CP_C_ of 2 articles, had the highest CPP_2024_ of 1870 and 2459 for single-country and internationally collaborative articles, respectively. Switzerland had the highest CPP_2024_ of 1615 for single-author articles (SP = 1).

Of the 42 classic articles, 18 (43%) were published by a single institution, achieving a CPP_2024_ of 1571 citations per publication. The remaining 24 articles (57%) resulted from inter-institutional collaborations, which had a lower CPP_2024_ of 1371. Similar to international collaborations, institutional collaborations were associated with a modest decrease in citation impact in this field. These findings suggest that, like international collaboration, institutional collaboration was associated with a modest decrease in citation impact within education-focused nursing research.

Supplementary table 2 presents publication data for the 9 most productive institutions, each with 3 or more classic articles. Six of these are from the USA, and 1 each from China, Sweden, and Switzerland. The University of North Carolina (USA) was the only institution to publish single-author classic articles listed in Supplementary table 2. Only the University of North Carolina and the University of Bern (Switzerland) produced single-institution classic articles. The University of Michigan and University of Washington (both USA) ranked highest with a TP of 6 articles 14% of the total), and a CP_I_ of 6 articles (total classic articles and internationally collaborative articles combined). Eighteen institutions contributed single-institution articles with an IP_I_ of 1 article (5.6% of single-institution articles). The University of Miami (USA) and University of Bern (Switzerland) ranked highest with FP of 2 articles (4.8% of 42) and RP of 2 articles (4.8% of 42), respectively. The University of Washington also had 2 first-author classic articles.

Nine institutions contributed single-author articles, with an SP of 1 article each (14% of 7 single-author articles). Among the top 9 institutions, the University of North Carolina (USA), with 4 total articles, had the highest CPP_2024_ across all categories, including:

• Total publications: 1643 citations.
• Single-institution publications: 1678 citations.
• Inter-institutional collaborative publications: 1631 citations.
• Single-author publications: 1678 citations.
• Corresponding-author publications: 1678 citations.

Several interrelated factors explain the strong research performance observed. Institutions with strong medical foundations, such as Karolinska Institutet (Sweden) and the University of North Carolina (USA), excel at producing impactful research, notably in dentistry.^61^, ^62^, ^63^, ^64^ These countries are also prominent in related fields like prosthodontics, endodontics, and temporomandibular disorders.^65^, ^66^, ^67^, ^68^ Their focus on interdisciplinary collaboration, investment in research infrastructure, and clinically relevant topics are key drivers of their high citation performance.^61^, ^62^, ^63^, ^64^

Sweden, in particular, has a tradition of prioritizing evidence-based practice in medicine, dentistry, and nursing,^69^, ^70^, ^71^ supported by robust academic infrastructure and quality-focused funding frameworks.^72^ Sweden’s research impact, notably via the University of Gothenburg and Karolinska Institutet, is strengthened by historical leadership in periodontal research, implantology, and temporomandibular disorders, fostering extensive international collaborations.^65^ Swedish institutions excel at translating basic research into clinical practice, influencing national and international guidelines. Karolinska Institutet contributes significantly, accounting for over 40% of Sweden’s academic medical and life sciences research output.^73^ The University of Gothenburg's high citation performance originates partly from Professor Brånemark’s pioneering osseointegration work in the 1960s, sustaining its global leadership in implant dentistry.^74^, ^75^, ^76^

The University of North Carolina’s strong performance in dentistry, oral surgery, and oral medicine stems from its long-standing innovative research tradition, especially in periodontal disease, oral epidemiology, and implantology. Its multidisciplinary approach integrates epidemiology, clinical dentistry, and public health, facilitating influential collaborations and research outputs.^61^^,^^62^ An example is the validated Oral Health Impact Profile (OHIP) by Slade.^59^

Together, these institutions effectively use interdisciplinary collaboration and research translation, bridging foundational science and clinical application, underpinning their exceptional global citation performance.

Among the 42 classic articles in the “dentistry, oral surgery and medicine” category of SCI-EXPANDED, the average number of authors per publication (APP) was 5.9, with a median of 3.0 authors. Most of the articles were collaborative efforts among small groups: 10 articles (24%) had 2 authors, 7 articles (17%) were single-author, 5 articles (12%) had 3 authors, and 4 articles (10%) involved 4 authors. In total, 62% of the classic articles were authored by groups of 1 to 4 authors. Only 1 article^12^ was published with multiple corresponding authors.

Table 3 presents the top 22 most productive classic authors, evaluated using 4 publication indicators, their corresponding citation impact (CPP_2024_), and Y-index constants.^65^ Key findings include:

**Table 3:** Top 22 productive authors with 2 or more classic articles.

Author	TP (n = 42 articles)		FP (n = 42 articles)		RP (n = 42 articles)		SP (n = 42 articles)		h (n = 42)	rank (j)
	rank (TP)	CPP_2024_	rank (FP)	CPP_2024_	rank (RP)	rank (RP)	rank (SP)	CPP_2024_		
P.I. Eke	1 (3)	1118	1 (2)	1123	1 (2)	1123	N/A	N/A	π/4	1 (4)
M.S. Tonetti	1 (3)	1320	5 (1)	1517	6 (1)	1517	N/A	N/A	π/4	6 (2)
K.S. Kornman	1 (3)	1320	N/A	N/A	N/A	N/A	N/A	N/A	0	43 (0)
B. Mehrotra	4 (2)	1509	N/A	N/A	N/A	N/A	N/A	N/A	0	43 (0)
B.A. Dye	4 (2)	1123	N/A	N/A	N/A	N/A	N/A	N/A	0	43 (0)
C.J.L. Murray	4 (2)	1105	N/A	N/A	N/A	N/A	N/A	N/A	0	43 (0)
D. Buser	4 (2)	1021	1 (2)	1021	1 (2)	1021	N/A	N/A	π/4	1 (4)
E. Bernabé	4 (2)	1105	N/A	N/A	N/A	N/A	N/A	N/A	0	43 (0)
G. Armitage	4 (2)	1118	N/A	N/A	N/A	N/A	N/A	N/A	0	43 (0)
G.D. Slade	4 (2)	1351	5 (1)	1678	6 (1)	1678	1 (1)	1678	π/4	6 (2)
G.O. Thornton-Evans	4 (2)	1123	N/A	N/A	N/A	N/A	N/A	N/A	0	43 (0)
H. Greenwell	4 (2)	1394	N/A	N/A	N/A	N/A	N/A	N/A	0	43 (0)
L. Wei	4 (2)	1123	N/A	N/A	N/A	N/A	N/A	N/A	0	43 (0)
M. Sanz	4 (2)	1222	N/A	N/A	N/A	N/A	N/A	N/A	0	43 (0)
N.J. Kassebaum	4 (2)	1105	5 (1)	1152	N/A	N/A	N/A	N/A	0	35 (1)
P.N. Papapanou	4 (2)	1222	5 (1)	1271	6 (1)	1271	N/A	N/A	π/4	6 (2)
R.C. Page	4 (2)	1065	5 (1)	1107	6 (1)	1107	N/A	N/A	π/4	6 (2)
R.E. Marx	4 (2)	1533	1 (2)	1533	1 (2)	1533	N/A	N/A	π/4	1 (4)
R.J. Genco	4 (2)	1123	N/A	N/A	N/A	N/A	N/A	N/A	0	43 (0)
S.L. Ruggiero	4 (2)	1509	1 (2)	1509	1 (2)	1509	N/A	N/A	π/4	1 (4)
T. Berglundh	4 (2)	1118	5 (1)	1064	6 (1)	1064	N/A	N/A	π/4	6 (2)
W. Marcenes	4 (2)	1105	5 (1)	1058	1 (2)	1105	N/A	N/A	1.107	5 (3)

TP, total number of classic articles; FP, first-author classic articles; RP, corresponding-author classic articles; CPP_2024_, average number of citations per publication (CPP_2024_ = TC_2024_/TP); *j*, a Y-index constant related to the publication potential; h, a Y-index constant related to the publication characteristics; N/A, not available.

P.I. Eke led in 3 indicators:

Total Publications (TP = 3), First-Author Publications (FP = 2), and Corresponding-Author Publications (RP = 2).

M.S. Tonetti and K.S. Kornman each also had TP = 3 classic articles.

R.E. Marx, S.L. Ruggiero, and D. Buser each authored 2 first-author articles (FP = 2).

R.E. Marx, W. Marcenes, S.L. Ruggiero, and D. Buser had 2 corresponding-author articles (RP = 2).

R.E. Marx, with 2 classic articles, recorded the highest CPP_2024_ among the top 22 authors: 1533 citations per publication. G.D. Slade was the only author to be a first-author, corresponding-author, and single-author on a classic article (FP = 1, RP = 1, SP = 1), with a CPP_2024_ of 1678 across all authorship roles.

Ten of these 22 authors, including Eke, Tonetti, Marx, Ruggiero, Buser, Marcenes, Slade, Papapanou, Page, and Berglundh, were also among the top 22 authors in cochlear implant research as measured by the Y-index, suggesting sustained impact across disciplines.

A total of 221 unique authors contributed to the 42 classic articles:

• 179 authors (81%) were not first or corresponding authors (Y-index = [0, 0]). Example: K.S. Kornman, with 3 classic articles, had no first- or corresponding-author roles.
• Four authors (1.8%) published only as corresponding authors (h = π/2): Q.M. Chen, S. Shi, S.L. Dohan, and T.W. Li, each with Y-index (1, π/2).
• One author (0.45%), W. Marcenes, had more corresponding-author than first-author roles (Y-index = (3, 1.107), where π/4 < h < π/2).
• 33 authors (15%) had equal numbers of first- and corresponding-author articles (h = π/4): Includes P.I. Eke, D. Buser, R.E. Marx, and S.L. Ruggiero, each with Y-index (4, π/4), indicating highest productivity (j = 4).
• Four authors (1.8%) were exclusively first authors (h = 0), with Y-index^1, 0^: Includes N.J. Kassebaum, H. Xu, J. Choukroun, and S. Gronthos.

**Fig. 2:**
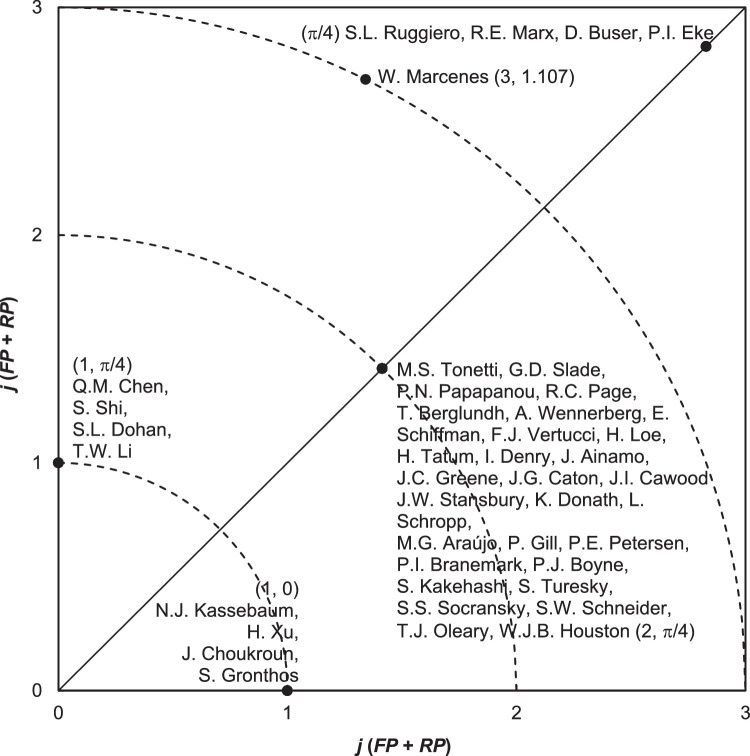
Distribution of the top 42 authors with Y-index (j ≥ 1).

Figure 2 illustrates the Y-index distribution for authors of the 42 classic articles. Each point represents a unique Y-index pair (j, h), where:

Authors on the same circular curve share the same publication potential (j). Authors along the same angular direction from the origin share the same authorship contribution ratio (h).

For example: P.I. Eke, D. Buser, R.E. Marx, and S.L. Ruggiero all share Y-index (4, π/4), highest potential and balanced roles as first and corresponding authors and T.W. Li (Y-index = (1, π/2)) and S. Gronthos (Y-index =^1, 0^) lie on the same j = 1 curve but differ in their authorship roles.

Authors along the diagonal h = π/4 (e.g., Eke and Tonetti) demonstrate equal contributions as first and corresponding authors, differing only in total output (j).

### Analysis of words in classic article title and author keywords

Word frequency analysis in article titles and author keywords is an effective method for identifying major research themes.^77^ Supplementary table 3 presents the 20 most frequently used words in titles and keywords of classic articles in “dentistry, oral surgery and medicine,” excluding predefined search terms. This lexical analysis helps reveal central topics and evolving areas of interest in the field.

The analysis shows a strong emphasis on periodontology and implant-related research among the most influential publications. In article titles, terms such as “bone,” “oral,” “classification,” “conditions,” and “periodontitis” are among the most frequently used, reflecting a consistent focus on structural and pathological aspects of oral tissues. The recurrence of “bone” (n = 7; 17%) and “oral” (n = 7; 17%) in titles illustrates the centrality of hard tissue biology and oral cavity-related conditions in classic literature.

The prominence of “classification” and “conditions” suggests a strong interest in standardizing diagnostic criteria and categorizing oral diseases, which is further echoed by keywords such as “case definition,” “classification,” and “periodontal diseases.” These results highlight the field’s long-standing efforts to refine clinical frameworks and improve diagnostic consistency.

Author keywords reinforce these patterns. “Periodontitis” appears most frequently (n = 7; 35%), followed by terms like “epidemiology,” “periodontal diseases,” “peri-implant mucositis,” and “peri-implantitis,” indicating the clinical importance and research focus on inflammatory and degenerative conditions affecting the periodontium and peri-implant tissues. This is consistent with the global burden of periodontal disease and the increasing clinical relevance of implant complications.

Other notable keywords include “surface topography,” “biomarkers,” “tooth loss,” and “3D printing,” suggesting emerging interest in biomaterial science, regenerative approaches, and advanced technologies in dental research. The presence of terms such as “osteonecrosis,” “acute periodontal conditions,” and “aggressive periodontitis” points to growing attention on high-risk patient groups and disease subtypes.

Altogether, the results from the word frequency analysis indicate that classic articles in the field of dentistry, oral surgery, and oral medicine predominantly focus on periodontology, implantology, disease classification, and diagnostic frameworks, while also reflecting increasing attention to technology-driven innovation and clinical outcomes

### Citation histories of the classic articles

Supplementary table 4 presents the 42 classic articles identified in the Web of Science category of ‘Dentistry, Oral Surgery and Medicine’ from the SCI-EXPANDED database. The citation trajectories of these articles, as illustrated in Supplementary figures 1 through 7, highlight notable trends in citation dynamics over time.

Several articles exhibited sharp citation increases shortly after publication, followed by a noticeable decline in recent years. Noteworthy examples include:

• Ruggiero et al.^43^ (Supplementary figure 2),
• Ruggiero et al.^78^ and Eke et al.^79^ (Supplementary figure 4),
• Marx et al.^80^ (Supplementary figure 6),
• Marcenes et al.^81^ (Supplementary figure 7).

These patterns may reflect the typical life cycle of influential research, rapid adoption and citation during their initial period of impact, followed by a gradual decrease as newer studies emerge, or the topic matures. The pronounced citation peaks observed in these classic articles, followed by a gradual decline, can be explained by clinical and scientific developments relevant to their subject matter. Articles such as Ruggiero et al.^43^^,^^78^ Marx et al.^80^ and Eke et al.^79^ exemplify this pattern. These works were published in response to pressing clinical challenges and contributed substantially to guideline development, which likely drove their initial citation surges.

For example, the 2 position papers by Ruggiero et al.^43^^,^^78^ provided the first definitions and clinical guidance related to medication-related osteonecrosis of the jaw, a complication primarily associated with bisphosphonate and antiresorptive therapies. The 2004 paper identified and described the phenomenon while the 2014 update expanded upon the initial criteria, reflecting the evolution of clinical experience and newer pharmacologic agents.^43^^,^^78^ Likewise, Marx et al. contributed histopathological validation of bisphosphonate-induced jaw necrosis, further solidifying the association and supporting changes in risk management practices.^80^ These publications were quickly integrated into clinical guidelines and became cornerstone references, which explains their rapid citation growth. However, as MRONJ became more widely understood and protocols stabilized, the academic need to reference these foundational papers diminished, resulting in the observed citation plateau.

Eke et al. similarly produced an impactful population-based epidemiological report on periodontitis in adults in USA. Its strength lay in the large-scale, standardized methodology adopted under CDC Periodontal Disease Surveillance Initiative guidance.^79^ This article initially gained broad traction in public health and periodontal literature but has since seen decreased citation activity as newer datasets and revisions in classification have emerged.

Taken together, these citation trajectories reflect a typical lifecycle of influential medical publications: rapid uptake due to clinical relevance and knowledge gaps, followed by a decline as the field matures or shifts. This pattern underscores the strong link between citation dynamics and the medical profession's evolving needs, regulatory developments, and practice transformations

Several classic articles in ‘dentistry, oral surgery and medicine’ have demonstrated sustained citation impact over extended periods, indicating their enduring influence in the field. For example:

• Ainamo and Bay^14^ and O’Leary et al.^82^ (Supplementary figure 1).
• Greene and Vermillion^57^ (Supplementary figure 2).
• Boyne and James^83^ (Supplementary figure 3).
• Kakehashi et al.^84^ (Supplementary figure 4).
• Schneider^85^ and Tatum^86^ (Supplementary figure 5).
• Turesky et al.^87^ and Vertucci^88^ (Supplementary figure 6).
• Cawood and Howell^89^ (Supplementary figure 7).

continue to receive notable citation counts as of 2024, highlighting their lasting relevance.

In contrast, some articles, despite a long citation history, have exhibited declining citation trends in recent years, such as:

• Donath and Breuner^90^ (Supplementary figure 2),
• Houston^91^ (Supplementary figure 5).

This variation suggests that while some foundational works remain consistently influential, others may experience a gradual reduction in attention as the field evolves or their contributions become absorbed into standard knowledge. A second group of classic articles within the field of 'Dentistry, Oral Surgery and Medicine' demonstrates sustained citation impact over several decades, underscoring their continued relevance and foundational role in dental science. Many of these works have introduced clinical techniques, indices, or anatomical insights that remain essential in practice and education. For instance, Ainamo and Bay’s article proposing a simplified method for recording plaque and gingivitis,^14^ and O’Leary et al.'s introduction of the plaque control record,^82^ both in Supplementary figure 1, have had a lasting impact due to their practical applicability in both clinical settings and research. These tools continue to be widely used in clinical trials and daily dental assessments, contributing to their continued citations.

Similarly, Greene and Vermillion’s work,^57^ featured in Supplementary figure 2, developed the Simplified Oral Hygiene Index (OHI-S), which has remained 1 of the most recognized and applied indices for evaluating oral cleanliness in population studies. The longevity of its citation profile illustrates the robustness and ease of use that researchers and clinicians alike continue to rely on.

In the domain of surgical techniques, Boyne and James^83^ described a bone grafting method for maxillary sinus floor augmentation, now considered a precursor to modern sinus lift procedures (Supplementary figure 3). Tatum,^86^ although not uploaded, is another pioneer in this domain, having elaborated on sinus augmentation techniques that underpin much of today’s implantology protocols. Their citation endurance likely stems from the fact that these procedures remain foundational in implant dentistry.

From a basic science perspective, Kakehashi et al.^84^ featured in Supplementary figure 4, established the pivotal role of microorganisms in pulp and periapical disease by using germ-free animal models, laying the groundwork for contemporary endodontic microbiology. Similarly, Vertucci’s detailed anatomical mapping of human root canal systems,^88^ represented in Supplementary figure 6, continues to be indispensable in endodontic education and clinical practice due to its clarity and comprehensiveness.

Diagnostic contributions have also endured. Schneider’s method for measuring canal curvature^85^ in Supplementary figure 5 is still frequently cited for its utility in anticipating procedural complexity in endodontics. Meanwhile, Turesky et al.^87^ as shown in Supplementary figure 6, refined existing plaque indices, enabling more reliable plaque quantification and further advancing preventive dentistry.

Finally, the classification of jaw atrophy patterns by Cawood and Howell^89^ in Supplementary figure 7, continues to inform pre-prosthetic and surgical planning. Their work’s ongoing relevance is tied to its simplicity and clinical usefulness in reconstructive procedures.

Together, these articles show how conceptual clarity, clinical applicability, and methodological robustness contribute to long-term scientific influence. Their persistent citation activity highlights that certain foundational works, despite the passage of time, remain cornerstones of dental science. This stands in contrast to other classics that, though initially impactful, have seen diminishing attention, often as their insights become integrated into the standard corpus of knowledge

Several articles have exhibited a sharp rise in citations shortly after publication, reflecting their immediate and strong impact within the ‘dentistry, oral surgery and medicine’ research community. These include:

• Schiffman et al.^11^ (Supplementary figure 1).
• Xu et al.^12^ (Supplementary figure 2).
• Tonetti et al.^92^ (Supplementary figure 3).
• Papapanou et al.^93^ (Supplementary figure 4).
• Caton et al.^94^, Kassebaum et al.^95^ and Stansbury and Idacavage^96^ (Supplementary figure 5).
• Gill et al.^97^ (Supplementary figure 6).
• Berglundh et al.^98^ (Supplementary figure 7).

These trends indicate that these studies achieved rapid recognition and influence following publication, often addressing emerging or highly relevant topics in the field. A third pattern evident in the citation trajectories of classic articles in the field of “Dentistry, Oral Surgery and Medicine” is the presence of a rapid surge in citations shortly after publication. This typically reflects the article’s immediate relevance to an emerging or widely debated topic, as well as its perceived authority and utility in clinical or research contexts. For example, Schiffman et al.^11^ introduced the DC/TMD, which replaced the earlier RDC/TMD. This publication (Supplementary figure 1) has gained considerable traction among clinicians and researchers due to its dual-axis biopsychosocial model, validated methodology, and global applicability, helping to standardize diagnostics in both clinical and research settings.

Similarly, Xu et al.^12^ represented in Supplementary figure 2, reviewed the relationship between oral and systemic inflammation. Their comprehensive analysis of oral dysbiosis and its implications for systemic diseases such as cardiovascular and metabolic disorders sparked widespread interest amid increasing awareness of oral-systemic health connections.

In 2018, a cluster of influential consensus reports and systematic reviews was published in the Journal of Clinical Periodontology, including Tonetti et al.,^92^ Papapanou et al.,^93^ and Caton et al.^94^ (Supplementary figures 3-5). These publications were part of the World Workshop on the Classification of Periodontal and Peri-Implant Diseases and Conditions. The revised classification system they proposed has since been globally adopted and cited extensively due to its relevance to both diagnosis and treatment planning.

Kassebaum et al.^95^ also included in Supplementary figure 5, contributed a global epidemiological perspective with data from the Global Burden of Disease Study, highlighting the prevalence and impact of oral diseases. Its value lies in providing authoritative global estimates and reinforcing oral health as a major public health priority.

The work by Stansbury and Idacavage^96^ provided a state-of-the-art overview of dental composite resins, a cornerstone material in restorative dentistry. Their article’s sharp rise in citations can be linked to both the importance of the topic and the clarity of their discussion on future trends in biomaterials.

Gill et al.^97^ featured in Supplementary figure 6, offered foundational guidance on qualitative research methodology in dentistry. As qualitative approaches have gained traction in dental public health and patient-centered care, this article remains a key reference for researchers adopting mixed-methods or interview-based designs.

Finally, the consensus report by Berglundh et al.^98^ on peri-implant diseases and conditions (Supplementary figure 7) has had a strong impact due to its clinical utility in differentiating between peri-implant mucositis and peri-implantitis, thus providing evidence-based guidance for prevention and treatment.

Together, these articles exemplify how clinical relevance, global applicability, and methodological rigor can drive rapid citation growth, particularly when aligned with emerging trends or international consensus efforts. Their early and steep citation curves illustrate a pattern of immediate integration into clinical practice, guideline development, and ongoing scholarly debate

Only 3 of the ten most frequently cited articles overall were also among the top ten most cited in the year 2024, underscoring the dynamic and evolving nature of scholarly impact in the ‘dentistry, oral surgery and medicine’ category. This discrepancy highlights how citation influence can shift over time, with some articles gaining renewed relevance while others decline. Key examples illustrating this trend are summarized below:

*1. Microbial complexes in subgingival plaque (Socransky et al.)*^8^

The article by Socransky et al. authored by 5 researchers from the Forsyth Dental Center, USA, achieved a TC_2024_ of 3470 citations, making it the most cited classic article in the ‘dentistry, oral surgery and medicine’ category, and ranked 6^th^ in citations for the year 2024 with C_2024_ of 222 citations. The study identified distinct bacterial complexes in subgingival plaque, with the Red complex notably exhibiting a strong correlation with clinical indicators of periodontitis. These findings significantly advanced understanding of the microbial ecology of periodontal disease, providing a foundation for improved diagnostic and therapeutic approaches. As shown in Supplementary figure 1, the article experienced a sharp rise in citations following its publication, reflecting its enduring influence and the substantial contribution of Socransky et al. to periodontal research.

*2. Diagnostic criteria for temporomandibular disorders (DC/TMD) for clinical and research applications: Recommendations of the international RDC/TMD consortium network and orofacial pain special interest group (Schiffman et al)*^11^

The internationally collaborative article by Schiffman et al. involved 34 authors from 25 institutions across 12 countries and achieved a TC_2024_ of 2599, ranking 2nd overall, and the most cited article in 2024 (C_2024_ of 475). This landmark study introduced the updated DC/TMD protocol, marking a significant advancement in the clinical classification and assessment of TMD. Axis I provides reliable diagnostic tools for pain-related TMDs and intra-articular disorders, while Axis II assesses psychosocial and functional factors through structured self-report instruments. Together, these evidence-based protocols represent a robust and standardized framework for both clinical and research applications, enhancing diagnostic accuracy, consistency, and patient care. The article has a steep and sustained increase in citations culminating in its top citation rank in 2024. Its strong citation growth indicates ongoing significant impact on TMD research and clinical practice (Supplementary figure 1).

*3. Problems and proposals for recording gingivitis and plaque (Ainamo and Bay)*^14^

Ainamo and Bay, by Professor Jukka Ainamo and Chief Dental Officer Inger Bay, proposed the Gingival Bleeding Index for efficiently recording gingival inflammation and plaque accumulation. The method provided an objective and reproducible indicator of gingivitis, significantly enhancing consistency in oral hygiene assessments. Although initially low-cited, citations surged notably from the mid-2000s, peaking at 179 in 2021 and maintaining high levels through 2024 (C2024: 147, Supplementary figure 1). This delayed increase reflects rising global emphasis on standardized periodontal assessments for diagnostics, treatment outcomes, and investigating systemic disease links, emphasizing the index’s continued practical relevance.^99^, ^100^, ^101^, ^102^, ^103^, ^104^, ^105^, ^106^, ^107^, ^108^, ^109^, ^110^

## Conclusion

This bibliometric analysis offers a comprehensive overview of the most highly cited publications in the field of dentistry, oral surgery, and medicine. It reveals not only the publication and citation patterns of classic articles but also highlights the contributions of specific countries, institutions, and authors. The findings suggest that long-term citation impact is often shaped by research quality, clinical applicability, and international relevance. While older articles tend to accumulate citations steadily, newer publications may experience rapid and intense attention, especially when addressing contemporary challenges or offering updated consensus guidelines. Institutions such as the University of North Carolina and Karolinska Institutet have achieved exceptional influence through sustained investment in interdisciplinary research and clinically impactful topics. These insights provide valuable context for future researchers and policymakers aiming to understand and foster scientific excellence within the dental sciences.

## Statement about originality

The research conducted in this manuscript is original, not presently under consideration for publication elsewhere and free of conflict of interest. Both authors alone are responsible for the content and writing of the paper.

## Ethics statement

Not applicable since data was publicly available

## Patient consent

Not applicable since no patient data is reported

## Availability of data and materials

Data generated and/or analyzed during the current study are available from the corresponding author on reasonable request.

## Artificial intelligence

No artificial intelligence has been used in any part of the analysis or writing.

## Authors' contributions

YH and NC contributed to the conceptualization, and methodology. YH contributed to data collection, data curation, and formal data analysis. YH and NC contributed and interpretation. NC wrote the original draft, while YH reviewed the manuscript. Finally, both authors read and revised the manuscript prior to submission.

## Funding

None.

## Conflict of interests

The authors of this work declare that they have no competing interests to disclose.

## Appendix Supplementary materials

**Supplementary figure 1.** Citation histories of the top 6 articles in the Web of Science category of dentistry, oral surgery and medicine Image, application 1

**Supplementary figure 2.** Citation histories of the top 7-12 articles in the Web of Science category of dentistry, oral surgery and medicine Image, application 2

**Supplementary figure 3.** Citation histories of the top 13-18 articles in the Web of Science category of dentistry, oral surgery and medicine Image, application 3

**Supplementary figure 4.** Citation histories of the top 19-24 articles in the Web of Science category of dentistry, oral surgery and medicine Image, application 4

**Supplementary figure 5.** Citation histories of the top 25-30 articles in the Web of Science category of dentistry, oral surgery and medicine Image, application 5

**Supplementary figure 6.** Citation histories of the top 31-36 articles in the Web of Science category of dentistry, oral surgery and medicine Image, application 6

**Supplementary figure 7.** Citation histories of the top 37-42 articles in the Web of Science category of dentistry, oral surgery and medicine Image, application 7

Image, application 8

Image, application 9

Image, application 10

Image, application 11

## Footnotes

Supplementary material associated with this article can be found in the online version at doi:10.1016/j.identj.2025.100909.
