# Supplementary material for: Classic Publications in the Field of Dentistry: A Bibliometric Analysis
Source: Int Dent J. 2025 Jul 17;75(5):100909. doi: 10.1016/j.identj.2025.100909 (PMC12284788; doi:10.1016/j.identj.2025.100909)
Supplement: Supplementary file 3 — Supplementary figure 3. Citation histories of the top 13-18 articles in the Web of Science category of dentistry, oral surgery and medicine [file mmc3.docx]

**Supplementary figure 3.** Citation histories of the top 13-18 articles in the Web of Science category of dentistry, oral surgery and medicine
