# Supplementary material for: Classic Publications in the Field of Dentistry: A Bibliometric Analysis
Source: Int Dent J. 2025 Jul 17;75(5):100909. doi: 10.1016/j.identj.2025.100909 (PMC12284788; doi:10.1016/j.identj.2025.100909)
Supplement: Supplementary file 8 [file mmc8.docx]

**Supplementary table 1.** Citations and authors according to the document type.

| Document type | TP | % | AU | APP | TC_2024_ | CPP_2024_ |
| --- | --- | --- | --- | --- | --- | --- |
| Article | 42 | 67 | 246 | 5.9 | 61,185 | 1,457 |
| Review | 18 | 29 | 88 | 4.9 | 29,784 | 1,655 |
| Proceedings paper | 4 | 6.3 | 58 | 15 | 4,956 | 1,239 |
| Editorial material | 1 | 1.6 | 2 | 2.0 | 1,081 | 1,081 |
| Letter | 1 | 1.6 | 1 | 1.0 | 2,016 | 2,016 |
| Meeting abstract | 1 | 1.6 | 1 | 1.0 | 2,640 | 2,640 |

TP: total number of classic publications; %: percentage of articles in all articles; AU: total number of authors; APP: average number of authors per publication; TC_2024_: total number of citations from WoSCC since publication year until the end of 2024; CPP_2024_: average number of citations per publication (TC_2024_/TP).
