# Supplementary material for: Classic Publications in the Field of Dentistry: A Bibliometric Analysis
Source: Int Dent J. 2025 Jul 17;75(5):100909. doi: 10.1016/j.identj.2025.100909 (PMC12284788; doi:10.1016/j.identj.2025.100909)
Supplement: Supplementary file 9 [file mmc9.docx]

**Supplementary table 2.** Top nine most productive institutions with three or more classic articles.

| Institution | TP | TP (n = 42) | | IP_I_ (n = 18) | | CP_I_ (n = 24) | | FP (n = 42) | | RP (n = 42) | | SP (n = 7) | |
| --- | --- | --- | --- | --- | --- | --- | --- | --- | --- | --- | --- | --- | --- |
|  |  | R (%) | CPP_2024_ | R (%) | CPP_2024_ | R (%) | CPP_2024_ | R (%) | CPP_2024_ | R (%) | CPP_2024_ | R (%) | CPP_2024_ |
| Univ Michigan | 6 | 1 (14) | 1,441 | N/A | N/A | 1 (25) | 1,441 | N/A | N/A | N/A | N/A | N/A | N/A |
| Univ Washington | 6 | 1 (14) | 1,429 | N/A | N/A | 1 (25) | 1,429 | 1 (4.8) | 1,130 | 3 (2.4) | 1,107 | N/A | N/A |
| SUNY Buffalo | 4 | 3 (10) | 1,529 | N/A | N/A | 3 (17) | 1,529 | N/A | N/A | N/A | N/A | N/A | N/A |
| UCSF | 4 | 3 (10) | 1,193 | N/A | N/A | 3 (17) | 1,193 | N/A | N/A | N/A | N/A | N/A | N/A |
| Univ Gothenburg | 4 | 3 (10) | 1,183 | N/A | N/A | 3 (17) | 1,183 | 4 (2.4) | 1,064 | 3 (2.4) | 1,064 | N/A | N/A |
| Univ N Carolina | 4 | 3 (10) | 1,643 | 1 (5.6) | 1678 | 6 (13) | 1,631 | 4 (2.4) | 1,678 | 3 (2.4) | 1,678 | 1 (14) | 1,678 |
| Columbia Univ | 3 | 7 (7.1) | 1,169 | N/A | N/A | 6 (13) | 1,169 | 4 (2.4) | 1,271 | 3 (2.4) | 1,271 | N/A | N/A |
| Univ Bern | 3 | 7 (7.1) | 1,035 | 1 (5.6) | 1034 | 9 (8.3) | 1,036 | 1 (4.8) | 1,021 | 1 (4.8) | 1,021 | N/A | N/A |
| Univ Hong Kong | 3 | 7 (7.1) | 1,320 | N/A | N/A | 6 (13) | 1,320 | 4 (2.4) | 1,517 | 3 (2.4) | 1,517 | N/A | N/A |

TP: total number of classic articles; TP R (%): total number of articles and percentage of total 42 articles; IP_I_ R (%): rank and percentage of single-institution articles in all 18 single-institution articles; CP_I_ R (%): rank and percentage of inter-institutionally collaborative articles in all 24 inter-institutionally collaborative articles; FP R (%): rank and percentage of first-author articles in all 42 first-author articles; RP R (%): rank and percentage of corresponding-author articles in all 42 corresponding-author articles; SP R (%): rank and the percentage of single-author articles in all 7 single-author articles; CPP_2024_: average number of citations per publication (CPP_2024_ = TC_2024_/TP); N/A: not available.

**Univ Michigan:** University of Michigan, USA; **Univ Washington:** University of Washington, USA; **University of Buffalo:** SUNY Buffalo, USA; **UCSF:** University of California, San Francisco, USA; **Univ Gothenburg:** University of Gothenburg, Sweden; **Univ N Carolina:** University of North Carolina, USA; **Columbia Univ:** Columbia University, USA; **Univ Bern:** University of Bern, Switzerland; **Univ Hong Kong:** University of Hong Kong, China
