# Supplementary material for: Classic Publications in the Field of Dentistry: A Bibliometric Analysis
Source: Int Dent J. 2025 Jul 17;75(5):100909. doi: 10.1016/j.identj.2025.100909 (PMC12284788; doi:10.1016/j.identj.2025.100909)
Supplement: Supplementary file 10 [file mmc10.docx]

**Supplementary table 3.** Top 20 most frequency used words in classic article title and author keywords.

| Words in title | *TP* | R (%) n = 42 | Author keywords | *TP* | R (%) n = 20 |
| --- | --- | --- | --- | --- | --- |
| Bone | 7 | 1 (17) | Periodontitis | 7 | 1 (35) |
| Oral | 7 | 1 (17) | Epidemiology | 4 | 2 (20) |
| Classification | 5 | 3 (12) | Periodontal diseases | 4 | 2 (20) |
| Conditions | 5 | 3 (12) | Periodontal disease | 3 | 4 (15) |
| Periodontitis | 5 | 3 (12) | Case definition | 2 | 5 (10) |
| Analysis | 4 | 6 (10) | Classification | 2 | 5 (10) |
| Diseases | 4 | 6 (10) | Necrotizing periodontitis | 2 | 5 (10) |
| Plaque | 4 | 6 (10) | Oral health | 2 | 5 (10) |
| Dental | 3 | 9 (7.1) | Peri-implant mucositis | 2 | 5 (10) |
| Effects | 3 | 9 (7.1) | Peri-implantitis | 2 | 5 (10) |
| Experimental | 3 | 9 (7.1) | Surface topography | 2 | 5 (10) |
| Global | 3 | 9 (7.1) | Tooth loss | 2 | 5 (10) |
| Jaws | 3 | 9 (7.1) | 3D printing | 1 | 13 (5) |
| Osteonecrosis | 3 | 9 (7.1) | 3Y-TZP | 1 | 13 (5) |
| Peri-implant | 3 | 9 (7.1) | Acute periodontal conditions | 1 | 13 (5) |
| Periodontal | 3 | 9 (7.1) | Additive manufacturing | 1 | 13 (5) |
| Prevalence | 3 | 9 (7.1) | Adults | 1 | 13 (5) |
| Report | 3 | 9 (7.1) | Aggressive periodontitis | 1 | 13 (5) |
| Sinus | 3 | 9 (7.1) | Bacteria | 1 | 13 (5) |
| Bone | 7 | 1 (17) | Biomarkers | 1 | 13 (5) |

TP: number of articles; %: percentage; R: rank.
