# Supplementary material for: Classic Publications in the Field of Dentistry: A Bibliometric Analysis
Source: Int Dent J. 2025 Jul 17;75(5):100909. doi: 10.1016/j.identj.2025.100909 (PMC12284788; doi:10.1016/j.identj.2025.100909)
Supplement: Supplementary file 11 [file mmc11.docx]

**Supplementary table 4.** 42 classic articles in the Web of Science category of ‘dentistry, oral surgery and medicine’

| Rank  (TC_2024_) | Rank  (C_2024_) | Title | Country | Reference |
| --- | --- | --- | --- | --- |
| 1 (3,470) | 6 (222) | Microbial complexes in subgingival plaque | USA | Socransky et al. (1998) (8) |
| 2 (2,599) | 1 (475) | Diagnostic Criteria for Temporomandibular Disorders (DC/TMD) for Clinical and Research Applications: Recommendations of the International RDC/TMD Consortium Network and Orofacial Pain Special Interest Group | USA, Canada, Sweden, Denmark, Netherlands, Italy, Switzerland, Germany, Belgium, Australia, France, UK | Schiffman et al. (2014) (11) |
| 3 (2,332) | 30 (63) | Experimental gingivitis in man | Denmark | Loe et al. (1965) (13) |
| 4 (2,319) | 9 (147) | Problems and proposals for recording gingivitis and plaque | Finland, Denmark | Ainamo and Bay (1975) (14) |
| 5 (1,980) | 11 (141) | Plaque control record | USA | Oleary et al. (1972) (82) |
| 6 (1,929) | 31 (60) | Platelet-rich plasma: Growth factor enhancement for bone grafts | USA | Marx et al. (1998) (58) |
| 7 (1,754) | 18 (94) | High expression of ACE2 receptor of 2019-nCoV on the epithelial cells of oral mucosa | China | Xu et al. (2020) (12) |
| 8 (1,678) | 13 (127) | Derivation and validation of a short-form oral health impact profile | USA | Slade (1997) (59) |
| 9 (1,633) | 14 (115) | American Association of Oral and Maxillofacial Surgeons Position Paper on Medication-Related Osteonecrosis of the Jaw-2014 Update | USA, Canada | Ruggiero et al. (2014) (43) |
| 10 (1,615) | 20 (84) | The World Oral Health Report 2003: continuous improvement of oral health in the 21st century: The approach of the WHO Global Oral Health Programme | Switzerland | Petersen (2003) (3) |
| 11 (1,592) | 39 (24) | A method for the study of undecalcified bones and teeth with attached soft tissues: The Säge-Schliff (sawing and grinding) technique | Germany | Donath and Breuner (1982) (90) |
| 12 (1,559) | 19 (85) | The simplified oral hygiene index | USA | Greene and Vermillion (1964) (57) |
| 13 (1,517) | 2 (385) | Staging and grading of periodontitis: Framework and proposal of a new classification and case definition | China, USA | Tonetti et al. (2018) (92) |
| 14 (1,513) | 35 (47) | Stem cell properties of human dental pulp stem cells | USA, UK | Gronthos et al. (2002) (111) |
| 15 (1,492) | 17 (98) | State of the art of zirconia for dental applications | USA | Denry and Kelly (2008) (112) |
| 16 (1,475) | 15 (109) | Dimensional ridge alterations following tooth extraction. An experimental study in the dog | Brazil, Sweden | Araújo and Lindhe (2005) (113) |
| 17 (1,464) | 29 (65) | Grafting of the maxillary sinus floor with autogenous marrow and bone | USA | Boyne and James (1980) (83) |
| 18 (1,407) | 21 (82) | Bone healing and soft tissue contour changes following single-tooth extraction: A clinical and radiographic 12-month prospective study | Denmark | Schropp et al. (2003) (114) |
| 19 (1,393) | 10 (144) | Platelet-rich fibrin (PRF): A second-generation platelet concentrate. Part V: Histologic evaluations of PRF effects on bone allograft maturation in sinus lift | France, USA, Sweden | Choukroun et al. (2006) (115) |
| 20 (1,385) | 40 (22) | Osteonecrosis of the jaws associated with the use of bispHosphonates: A review of 63 cases | USA | Ruggiero et al. (2004) (78) |
| 21 (1,324) | 25 (68) | Osseointegration and its experimental background | Canada, Sweden | Branemark (1983) (9) |
| 22 (1,291) | 36 (46) | The effects of surgical exposures of dental pulps in germ-free and conventional laboratory rats | USA | Kakehashi et al. (1965) (84) |
| 23 (1,271) | 3 (270) | Periodontitis: Consensus report of workgroup 2 of the 2017 World Workshop on the Classification of Periodontal and Peri-Implant Diseases and Conditions | USA, Spain, Turkey, UK, Brazil, China, Italy, Germany, Netherlands, Israel, Switzerland, Australia | Papapanou et al. (2018) (93) |
| 24 (1,223) | 24 (71) | Prevalence of Periodontitis in Adults in the United States: 2009 and 2010 | USA | Eke et al. (2012) (79) |
| 25 (1,218) | 22 (80) | Comparison of canal preparations in straight and curved root canals | USA | Schneider (1971) (85) |
| 26 (1,172) | 4 (252) | A new classification scheme for periodontal and peri-implant diseases and conditions: Introduction and key changes from the 1999 classification | USA, Sweden, UK, Germany, Spain, China | Caton et al. (2018) (94) |
| 27 (1,171) | 38 (25) | The analysis of errors in orthodontic measurements | UK | Houston (1983) (91) |
| 28 (1,169) | 32 (58) | Maxillary and sinus implant reconstructions | USA | Tatum (1986) (86) |
| 29 (1,152) | 7 (187) | Global, regional, and national prevalence, incidence, and disability-adjusted life years for oral conditions for 195 countries, 1990-2015: A systematic analysis for the global burden of diseases, injuries, and risk factors | USA, UK | Kassebaum et al. (2017) (95) |
| 30 (1,143) | 8 (154) | 3D printing with polymers: Challenges among expanding options and opportunities | USA | Stansbury and Idacavage (2016) (96) |
| 31 (1,137) | 42 (17) | BispHosphonate-induced exposed bone (osteonecrosis/osteopetrosis) of the jaws: Risk factors, recognition, prevention, and treatment | USA, Canada | Marx et al. (2005) (80) |
| 32 (1,135) | 12 (140) | Methods of data collection in qualitative research: interviews and focus groups | UK | Gill et al. (2008) (97) |
| 33 (1,120) | 32 (58) | Reduced plaque formation by the chloromethyl analogue of victamine C | USA | Turesky et al. (1970) (87) |
| 34 (1,107) | 27 (67) | Case definitions for use in population: Based surveillance of periodontitis | USA | Page and Eke (2007) (116) |
| 35 (1,104) | 27 (67) | Effects of titanium surface topography on bone integration: a systematic review | Sweden | Wennerberg and Albrektsson (2009) (117) |
| 36 (1,095) | 22 (80) | Root canal anatomy of the human permanent teeth | USA | Vertucci (1984) (88) |
| 37 (1,064) | 5 (234) | Peri-implant diseases and conditions: Consensus report of workgroup 4 of the 2017 World Workshop on the Classification of Periodontal and Peri-Implant Diseases and Conditions | Sweden, USA, Brazil, Spain, Australia, Switzerland, South Korea, Belgium, Germany | Berglundh et al. (2018) (98) |
| 38 (1,061) | 34 (49) | A classification of the edentulous jaws | UK | Cawood and Howell (1988) (89) |
| 39 (1,058) | 25 (68) | Global Burden of Oral Conditions in 1990-2010: A Systematic Analysis | UK, USA, Australia | Marcenes et al. (2013) (81) |
| 40 (1,034) | 41 (19) | Long-term evaluation of non-submerged ITI implants. Part 1: 8-year life table analysis of a prospective multi-center study with 2359 implants | Switzerland | Buser et al. (1997) (118) |
| 41 (1,023) | 16 (103) | Update on Prevalence of Periodontitis in Adults in the United States: NHANES 2009 to 2012 | USA | Eke et al. (2015) (119) |
| 42 (1,007) | 37 (42) | Enhanced bone apposition to a chemically modified SLA titanium surface | Switzerland, USA | Buser et al. (2004) (120) |

TC_2024_: the total number of citations from Web of Science Core Collection since the publication year until the end of 2024; C_2024_: the number of citations of an article in 2024 only.

**References**

3. Petersen PE. The World Oral Health Report 2003: continuous improvement of oral health in the 21st century--the approach of the WHO Global Oral Health Programme. Community Dent Oral Epidemiol. 2003;31 Suppl 1:3-23.

8. Socransky SS, Haffajee AD, Cugini MA, Smith C, Kent RL, Jr. Microbial complexes in subgingival plaque. J Clin Periodontol. 1998;25(2):134-44.

9. Brånemark PI. Osseointegration and its experimental background. J Prosthet Dent. 1983;50(3):399-410.

11. Schiffman E, Ohrbach R, Truelove E, Look J, Anderson G, Goulet JP, et al. Diagnostic Criteria for Temporomandibular Disorders (DC/TMD) for Clinical and Research Applications: recommendations of the International RDC/TMD Consortium Network* and Orofacial Pain Special Interest Group†. J Oral Facial Pain Headache. 2014;28(1):6-27.

12. Xu H, Zhong L, Deng J, Peng J, Dan H, Zeng X, et al. High expression of ACE2 receptor of 2019-nCoV on the epithelial cells of oral mucosa. International Journal of Oral Science. 2020;12(1):8.

13. Löe H, Theilade E, Jensen SB. Experimental Gingivitis in Man. The Journal of Periodontology. 1965;36(3):177-87.

14. Ainamo J, Bay I. Problems and proposals for recording gingivitis and plaque. Int Dent J. 1975;25(4):229-35.

43. Ruggiero SL, Dodson TB, Fantasia J, Goodday R, Aghaloo T, Mehrotra B, et al. American Association of Oral and Maxillofacial Surgeons position paper on medication-related osteonecrosis of the jaw—2014 update. Journal of oral and maxillofacial surgery. 2014;72(10):1938-56.

57. Greene JG, Vermillion JR. The simplified oral hygiene index. The Journal of the American Dental Association. 1964;68(1):7-13.

58. Marx RE, Carlson ER, Eichstaedt RM, Schimmele SR, Strauss JE, Georgeff KR. Platelet-rich plasma: Growth factor enhancement for bone grafts. Oral Surgery, Oral Medicine, Oral Pathology, Oral Radiology, and Endodontology. 1998;85(6):638-46.

59. Slade GD. Derivation and validation of a short‐form oral health impact profile. Community dentistry and oral epidemiology. 1997;25(4):284-90.

78. Ruggiero SL, Mehrotra B, Rosenberg TJ, Engroff SL. Osteonecrosis of the jaws associated with the use of bisphosphonates: a review of 63 cases. J Oral Maxillofac Surg. 2004;62(5):527-34.

79. Eke PI, Dye BA, Wei L, Thornton-Evans GO, Genco RJ. Prevalence of periodontitis in adults in the United States: 2009 and 2010. J Dent Res. 2012;91(10):914-20.

80. Marx RE, Sawatari Y, Fortin M, Broumand V. Bisphosphonate-induced exposed bone (osteonecrosis/osteopetrosis) of the jaws: risk factors, recognition, prevention, and treatment. J Oral Maxillofac Surg. 2005;63(11):1567-75.

81. Marcenes W, Kassebaum NJ, Bernabé E, Flaxman A, Naghavi M, Lopez A, et al. Global burden of oral conditions in 1990-2010: a systematic analysis. J Dent Res. 2013;92(7):592-7.

82. O'Leary TJ, Drake RB, Naylor JE. The plaque control record. J Periodontol. 1972;43(1):38.

83. Boyne PJ, James RA. Grafting of the maxillary sinus floor with autogenous marrow and bone. J Oral Surg. 1980;38(8):613-6.

84. Kakehashi S, Stanley HR, Fitzgerald RJ. The effects of surgical exposures of dental pulps in germ-free and conventional laboratory rats. Oral Surgery, Oral Medicine, Oral Pathology. 1965;20(3):340-9.

85. Schneider SW. A comparison of canal preparations in straight and curved root canals. Oral Surg Oral Med Oral Pathol. 1971;32(2):271-5.

86. Tatum H, Jr. Maxillary and sinus implant reconstructions. Dent Clin North Am. 1986;30(2):207-29.

87. Turesky S, Gilmore ND, Glickman I. Reduced plaque formation by the chloromethyl analogue of victamine C. J Periodontol. 1970;41(1):41-3.

88. Vertucci FJ. Root canal anatomy of the human permanent teeth. Oral Surg Oral Med Oral Pathol. 1984;58(5):589-99.

89. Cawood JI, Howell RA. A classification of the edentulous jaws. Int J Oral Maxillofac Surg. 1988;17(4):232-6.

90. Donath K, Breuner G. A method for the study of undecalcified bones and teeth with attached soft tissues. Journal of Oral Pathology & Medicine. 1982;11(4):318-26.

91. Houston WJ. The analysis of errors in orthodontic measurements. Am J Orthod. 1983;83(5):382-90.

92. Tonetti MS, Greenwell H, Kornman KS. Staging and grading of periodontitis: Framework and proposal of a new classification and case definition. J Periodontol. 2018;89 Suppl 1:S159-s72.

93. Papapanou PN, Sanz M, Buduneli N, Dietrich T, Feres M, Fine DH, et al. Periodontitis: Consensus report of workgroup 2 of the 2017 World Workshop on the Classification of Periodontal and Peri-Implant Diseases and Conditions. J Periodontol. 2018;89 Suppl 1:S173-s82.

94. Caton JG, Armitage G, Berglundh T, Chapple ILC, Jepsen S, Kornman KS, et al. A new classification scheme for periodontal and peri-implant diseases and conditions - Introduction and key changes from the 1999 classification. J Clin Periodontol. 2018;45 Suppl 20:S1-s8.

95. Kassebaum NJ, Smith AGC, Bernabé E, Fleming TD, Reynolds AE, Vos T, et al. Global, Regional, and National Prevalence, Incidence, and Disability-Adjusted Life Years for Oral Conditions for 195 Countries, 1990-2015: A Systematic Analysis for the Global Burden of Diseases, Injuries, and Risk Factors. J Dent Res. 2017;96(4):380-7.

96. Stansbury JW, Idacavage MJ. 3D printing with polymers: Challenges among expanding options and opportunities. Dent Mater. 2016;32(1):54-64.

97. Gill P, Stewart K, Treasure E, Chadwick B. Methods of data collection in qualitative research: interviews and focus groups. Br Dent J. 2008;204(6):291-5.

98. Berglundh T, Armitage G, Araujo MG, Avila-Ortiz G, Blanco J, Camargo PM, et al. Peri-implant diseases and conditions: Consensus report of workgroup 4 of the 2017 World Workshop on the Classification of Periodontal and Peri-Implant Diseases and Conditions. J Periodontol. 2018;89 Suppl 1:S313-s8.

111. Gronthos S, Brahim J, Li W, Fisher LW, Cherman N, Boyde A, et al. Stem cell properties of human dental pulp stem cells. J Dent Res. 2002;81(8):531-5.

112. Denry I, Kelly JR. State of the art of zirconia for dental applications. Dent Mater. 2008;24(3):299-307.

113. Araújo MG, Lindhe J. Dimensional ridge alterations following tooth extraction. An experimental study in the dog. J Clin Periodontol. 2005;32(2):212-8.

114. Schropp L, Wenzel A, Kostopoulos L, Karring T. Bone healing and soft tissue contour changes following single-tooth extraction: a clinical and radiographic 12-month prospective study. Int J Periodontics Restorative Dent. 2003;23(4):313-23.

115. Choukroun J, Diss A, Simonpieri A, Girard MO, Schoeffler C, Dohan SL, et al. Platelet-rich fibrin (PRF): a second-generation platelet concentrate. Part IV: clinical effects on tissue healing. Oral Surg Oral Med Oral Pathol Oral Radiol Endod. 2006;101(3):e56-60.

116. Page RC, Eke PI. Case definitions for use in population-based surveillance of periodontitis. J Periodontol. 2007;78(7 Suppl):1387-99.

117. Wennerberg A, Albrektsson T. Effects of titanium surface topography on bone integration: a systematic review. Clin Oral Implants Res. 2009;20 Suppl 4:172-84.

118. Buser D, Mericske-Stern R, Bernard JP, Behneke A, Behneke N, Hirt HP, et al. Long-term evaluation of non-submerged ITI implants. Part 1: 8-year life table analysis of a prospective multi-center study with 2359 implants. Clin Oral Implants Res. 1997;8(3):161-72.

119. Eke PI, Dye BA, Wei L, Slade GD, Thornton-Evans GO, Borgnakke WS, et al. Update on Prevalence of Periodontitis in Adults in the United States: NHANES 2009 to 2012. J Periodontol. 2015;86(5):611-22.

120. Buser D, Broggini N, Wieland M, Schenk RK, Denzer AJ, Cochran DL, et al. Enhanced bone apposition to a chemically modified SLA titanium surface. J Dent Res. 2004;83(7):529-33.
